# Home self-testing of complete blood counts in patients with breast cancer during chemotherapy: A proof-of-concept cohort study in e-oncology

**DOI:** 10.2340/1651-226X.2024.41050

**Published:** 2024-09-18

**Authors:** Lennart Friis-Hansen, Pippi Jonassen Bjørck, Ditte Hartvig, Susanne Andresen, Berit Rasmussen, Christina Hansen, Anne Nistrup, Keld Hundewadt, Niels Henrik Holländer

**Affiliations:** a Department of Clinical Oncology and Palliative Care, Zealand University Hospital, Næstved, Denmark; b Department of Clinical Biochemistry, Copenhagen University Hospital, Bispebjerg, Copenhagen, Denmark; c Department of Clinical Research, University of Southern Denmark, Odense, Denmark; d Department of Research, Zealand University Hospital, Køge, Denmark; e Department of Clinical Biochemistry, Zealand University Hospital, Køge, Denmark

**Keywords:** Home self-testing, complete blood count, point of care technology, breast cancer, self-monitoring, patient empowerment, patient education

## Abstract

**Background:**

Before administration of myelosuppressive chemotherapy, complete blood counts (CBC) collected at the hospital/nursing stations are evaluated to avoid severe bone marrow suppression. This maintains disease fixation which often reduces their quality of life. This mixed-method study examined at home self-testing of CBC, the test quality, and the effects on patients’ mental well-being.

**Methods:**

Patients with breast cancer receiving chemotherapy were recruited and trained to perform capillary finger prick CBC testing at home using the HemoScreen Point-of-Care instrument and to upload the test results to the hospital’s IT system subsequently. A venous reference CBC sample was taken and tested at the hospital on the day of self-testing. Semi-structured interviews with open-ended components were performed to investigate the user experience and the impact of self-testing on the patients’ everyday lives.

**Results:**

Thirty-nine patients completed the self-testing education using the HemoScreen instrument. Eight patients withdrew, while the remaining 31 patients performed 161 home tests (2–11 tests per patient) over a 4-month period. The test results compared well with the venous reference CBCs except for platelet counts (correlation coefficient 0.26). Qualitative interviews with nine of the 31 patients emphasized that the patients were comfortable using the self-testing instrument and becoming an active partner in their own treatment.

**Interpretation:**

CBC self-testing at home produced clinically valid hemoglobin and white blood cell counts with the added benefit that the patients became active partners in their own treatment course, which was of great importance for the patients and increased their wellbeing.

## Graphical abstract

**Figure:**
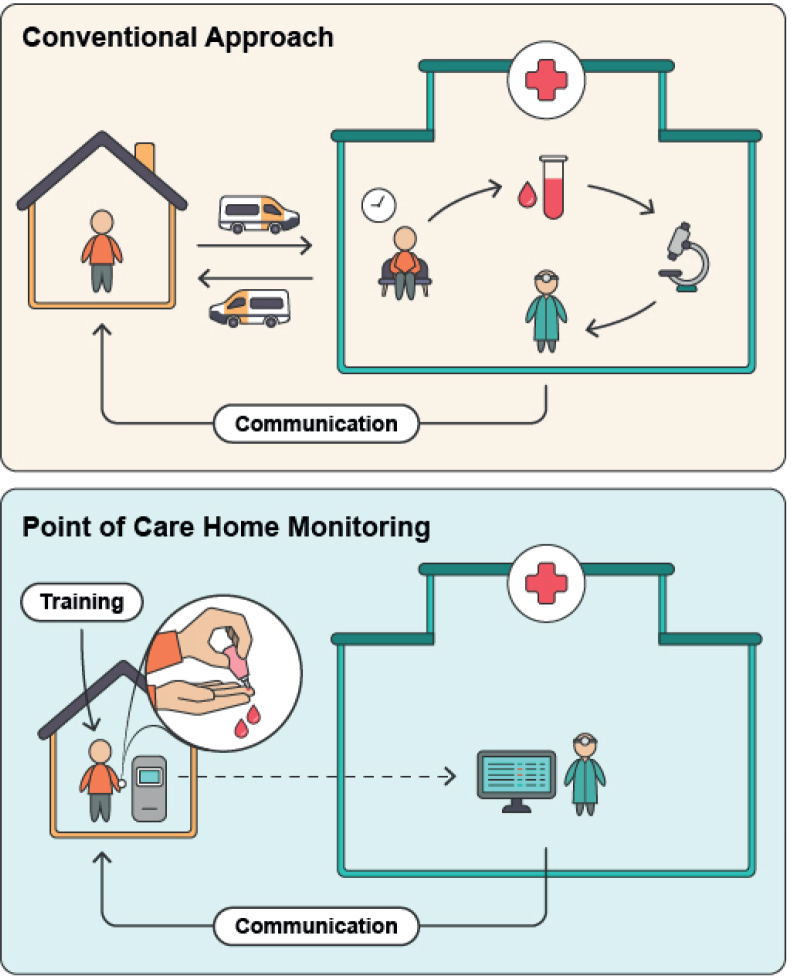

## Introduction

The regular monitoring and the supportive care needed to support safe antineoplastic treatment are resource-demanding for patients and healthcare professionals and furthermore associated with significant emotional stress among patients, their relatives, and caregivers. The multiple visits and long waiting time in outpatient clinics take up a considerable part of the patients’ time and interrupt their daily activities, often adding to the stress caused by the disease itself and lowering their quality of life [1]. Patient involvement and self-test/self-monitoring of several chronic diseases, including self-testing of biochemical parameters, has become an integrated part in the management of many chronic, for example, diabetes [2–4], vitamin K antagonist anticoagulation therapy [5], inflammatory bowel disease [6] and chronic obstructive pulmonary disease [7]. Patients with cancer, who gradually become active participants and co-designers of their treatment process, feel more empowered and have reduced disease-associated stress, and a better quality of life [8, 9]. However, so far, the focus of patient involvement in oncology has centered on patient-reported outcomes (PROs), video consultations and infusion of chemotherapy at home [10].

More than 5,000 women are yearly diagnosed with breast cancer in Denmark [11], and are treated with a combination of surgery, antihormone therapy, radiotherapy, and/or chemotherapy according to national guidelines; all scheduled over an extended period [12]. Outpatient clinics administer and follow up on the patient’s receiving chemotherapy which requires multiple visits for blood tests, physical evaluations, and treatment [13]. Besides the scheduled visits, acute hospitalizations may be required for treating acute infections due to febrile neutropenia (FN) due to high-dose chemotherapy-induced myelosuppression [14]. Twelve percent of patients treated for breast cancer in Denmark experience one or more episodes of FN [14]. To avoid severe therapy-induced myelosuppression, the bone marrow function is evaluated using a complete blood count (CBC) before administration of each cycle of chemotherapy, and based on the test results, the planned dose can be administered, reduced, or postponed [15]. Blood sampling is often performed the day before scheduled chemotherapy to ensure that the CBC and standard blood tests are available at the time of the outpatient oncology visit [15].

The quality of venous white blood cell counts (WBCs) and CBCs from point-of-care test (POCT) instruments meets the clinical requirements for monitoring CBC in patients with cancer receiving chemotherapy [16, 17]. Patients with cancer, regardless of age, can learn to operate POCT instruments satisfactorily during their visits to the outpatient clinic [16, 17]. However, for patients and caregivers to harvest the full benefit of self-testing and later self-monitoring [18], the POCT tests should be performed in the patients’ home, and the test results should be electronically transferred to the hospital records [19]. Therefore, we wanted to examine the performance of the complete CBC self-testing setup from patient training, instrument installation at home, instrument usability, test quality of capillary CBCs performed by the patients and transfer of test results from home to hospital. Furthermore, we wanted to describe how the home testing affected the patients’ wellbeing, learn their views on the setup’s benefits and drawbacks, their overall acceptance of the setup and learn how it impacted their role as patients.

## Materials and methods

### Study design and ethics

This single-center mixed method study tested the quality of the CBC test performed by the patients in their homes and examined how patients with breast cancer experienced performing the pre-therapy self-testing of CBCs at home. The study protocol (ClinicalTrials.gov NCT04543734) was approved by the Danish Data Protection Agency (REG-077-2020) and the Region Zealand’s Ethical Committee (ID SJ-840 - 91104).

### Setting and participants

The study was open to all patients with breast cancer aged 18 years or older with newly diagnosed breast cancer and planned for adjuvant chemotherapy treatment at the outpatient clinic at the Department of Oncology, Zealand University Hospital, Næstved, Denmark from December 20, 2020, to December 31, 2022. The patients were recruited at their first visit before the chemotherapy course was initiated (baseline) and would remain in the study until the end of treatment, approximately 3–4 months. Exclusion criteria were not understanding, reading, and speaking Danish or being unable to handle the test materials (i.e. lancet, sampler or cartridge) due to, for example, physical impairment such as paresthesia and muscle weakness in the hands. All participants were informed of their rights, including the possibility to withdraw from the study at any time, before giving informed consent. The study was initially set to include 33 patients (see power calculation, suppl info), but due to a software upgrade of the HemoScreen, the study was expanded to include 39 patients to allow for testing the impact of the software upgrade.

### Data collection and data handling

The patients’ demographic characteristics, tumor stage and type, performance status, and laboratory test results were recorded in a secure, dedicated SharePoint database.

### The HemoScreen hematology POCT instrument

The HemoScreen POCT CBC instrument (PixCell Medical, Yokne’am Illit, Israel) was chosen for the home self-testing based on our own [16] and others observations of its performance [20–22]. According to the European Union In Vitro Diagnostics Regulation (IVDR) Class C the instrument can only be operated by healthcare professionals; therefore, an insurance relieving the manufacturer of liabilities from damages resulting from off-label use was taken out.

### Patient education in HemoScreen CBC self-testing

One hour of education was allocated for teaching each patient how to collect the finger prick blood samples and operate the HemoScreen instrument. In brief, the patients were given a simple one-page stepwise illustrated guide to the test procedure. Next, the project nurse demonstrated the procedure, from finger prick test to data sharing in the electronic health record. To become certified, the patients had to complete the entire procedure themselves. The entire education including the patients self-test was observed, and a member of the research team scored the performance on a performance scale from 1 to 10, where 1 was extremely low and 10 stated excellent performance. To support the patients at home, they were given a one-page illustrative guide, access to a short ‘how to’ video, and a telephone hotline (see Supplementary Material).

### Verification of the capillary home self-test results

In the morning prior to the outpatient visits, the patients performed a capillary CBC home-test. The capillary CBC results were verified in the outpatient clinic <3 h after collections of the capillary home test by collecting a venous CBC reference blood sample which was analyzed within 1 h. after collection on the hospital’s XN-9000 analyzers (Sysmex, Corporation, Kobe, Japan). The venous CBC was considered the ‘gold standard’.

### Electronic transfer to the electronic health record

The hospital’s IT safety regulations prevented an end-to-end IT connection from the instrument to the laboratory information system. Instead, the patients were asked to take a picture of the HemoScreen test result using their cell phones and subsequently forward these to the hospital’s electronic health record software (Epic, Verona, WI) via the Danish version of the Epic App (‘MinSP’). This made the CBC test results available for the health care professional responsible for the patient’s care.

### Qualitative interview

The qualitative interviews were planned to include at least seven patients and continue enrolment until data saturation had been achieved defined as the point where three consecutive patients mentioned no new information [23]. The individual interviews were conducted at the patients’ homes when the patients were at least halfway through the planned chemotherapy cycles. The interviews followed a semi-structured interview guide consisting of open-ended questions about the feasibility and usability of the HemoScreen instrument and patients’ reflections on the challenges as well as the potentials related to self-testing of blood tests (see the interview guide, Supplementary information). To facilitate the interview, the patient and interviewer drew a timeline on paper detailing the perceived impact points from the patient’s time of diagnosis through early test and treatment until the interview date. The timeline was used as a reflective tool and a way of starting and structuring the dialogue related to the experiences with the HemoScreen instrument. Before the interview, the patient was asked to perform a self-test while being observed and scored by the interviewer. The interviews were recorded, fully or partly transcribed, and thematically analyzed [24].

### Statistical methods

The coefficient of variation (CV) for the HemoScreen analyzer, Deming, Bland-Altman and the correlation between the two analytical instruments were calculated with GraphPad Prism 9.5.1 (GraphPad Software, Boston, MA, USA).

## Results

### Recruitment and demography of patients for home monitoring

Thirty-nine high performance score (Karnofsky score 0–1) patients were recruited and completed the education: The median age was 52 years. The median distance from the patients’ homes to the Department of Oncology was 49 km, travel time by car 48 min while the median distance to the nearest blood sampling point was 11 km, travel time 10 min, Table 1. Eight of the 39 patients who completed the education later withdrew from the study: Two for medical reasons, three for technical reasons, and three for emotional reasons (Table 2). This did not change the overall characteristics of the cohort (Table 1).

**Table 1:** Demographics for patients in the study.

Parameter	All	Completed	Interviewed
Number of patients	39	31	9
Age	52 (27–70) years	52 (27–70) years	48 (27–58) years
Distance to oncology outpatient clinic	49 (5–103) km	48 (5–103) km	48 (5–58) km
Travel time to oncology outpatient clinic	48 (10–76) min	48 (10–76) min	43 (10–59) min
Distance to nearest blood sampling unit	11 (1–28) km	10 (1–28) km	8 (1–18) km
Travel time to nearest blood sampling unit	10 (1–31) min	10 (1–31)	6 (1–15) min
T-stage (T1/T2/T3)	23/12/4	19/8/4	2/5/2
N stage (N0/N1/N3)	26/12/1	21/9/1	5/3/1
M stage (M0)	39	31	9

The characteristics of the three groups in the study. All of those who volunteered (All), those who completed (Completed) the home monitoring study and those who were interview for the qualitative part of the study (Interviewed). When appropriate the results are given as median and range. Travel time is minutes by car.

**Table 2:** Reasons for withdrawing from the study.

**Medical reasons – two patients**
One patient withdrew due to development of fungal infection on hands which was incompatible with finger prick testing One patient withdrew due to hospitalization in relation to the cancer
**Technical – three patients**
One patient withdrew since she could not get instruments to work (had 3 different instruments) Although one patient did not respond to any communication from the staff, she informed them that she had not completed any tests during her chemotherapy. One patient withdrew after one test and noted the healthcare staff that she was not able to get enough blood for the test
**Emotional reason (did not like the concept) – three patients**
Two patients completed education but withdrew before they were handed an instrument as they had second thoughts about participating One patient withdrew and told that it was too much for her.

Description of the reasons for why eight participants withdrew from the study.

### The results of patient education

Half of the patients (19) were trained in the outpatient clinic while the other half (19) were trained in their own homes. All 39 patients who volunteered for the study satisfactorily completed the education including operating the instrument and transfering the test results using a cell phone; the median patient education score was 9 (6–10) (Table 3). Between 45 min and 1.5 hrs. were used to educate each participant.

**Table 3:** Patient self-testing scores and observations.

Parameter	All	Completed	Interviewed
Could complete the testing without help/	91%	89%	100%
Was confident in performing the test without supervision	95%	95%	100%
Could perform the test at home	100%	100%	100%
Education score	9 (6–10)	9 (6–10)	9 (7–10)
How was your overall experience	2 (1–2)	2 (1–2)	2 (1–2)
How would you rate the instruction	2 (2–2)	2 (2–2)	2 (2–2)
Confidence in performing the test after the instruction	100%	100%	100%
Able to perform the test at home after the instruction	100%	100%	100%
Like to do self-test at home	100%	100%	100%
Previously done finger prick blood sampling	14%	11%	0%
Self-tests performed at home	4 (0–11)	5 (2–11)	6 (4–11)
Days between 1st and last self-test at home	85 (14–134)	85 (14–134)	91 (71–132)
Test error/failure rate	0 (0–3)	0 (0–3)	0 (0–3)

Performance score (education score) of self-test and patients answer to post-test questions after completing the education session. All of those who volunteered (All), those who completed (Completed) the home monitoring and those who were interview for the qualitative part of the study (Interviewed). The results are given as percent or scored on a scale from 0 to 2 where 0 = not good and 2 = excellent. When appropriate the results are given as median and range.

### The performance of the capillary home self-testing

**Figure 1:**
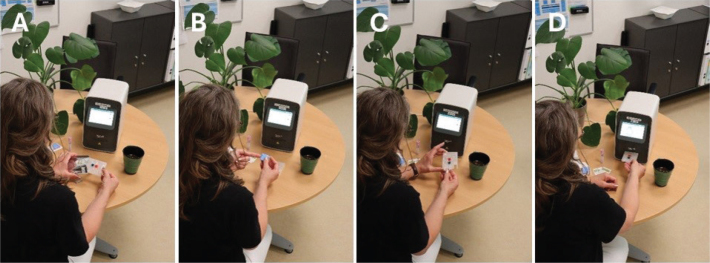
The home testing procedure using the HemoScreen instrument. (a) Preparing the test cartridge, (b) Collection of the capillary blood sample (‘fingerprick’), (c) Assembly of the test cartridge and (d) Loading the test cartridge into the instrument (model photo).

**Figure 2:**
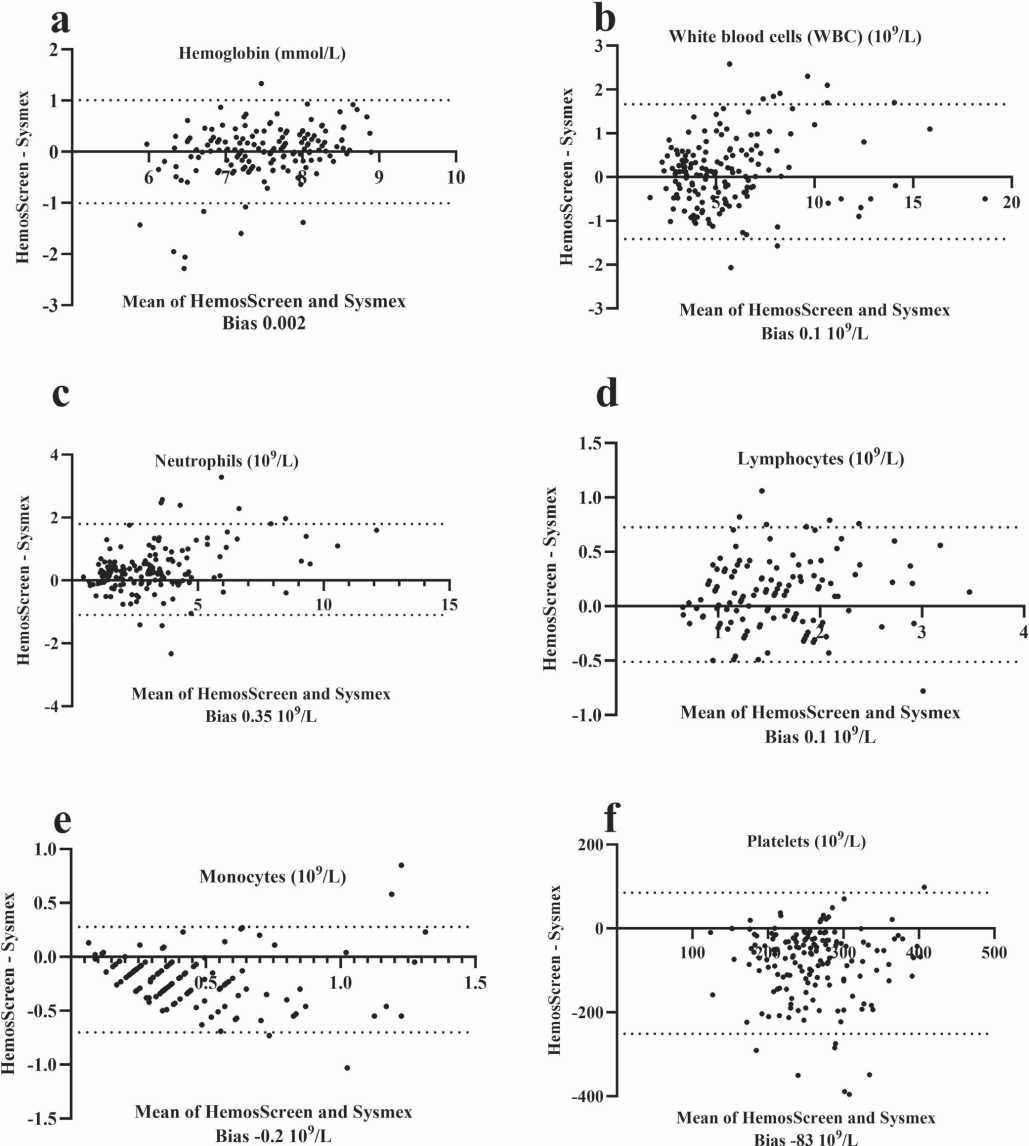
Bland-Altmann plot for patient collected capillary CBC analyzed using the HemoScreen compared with routine venous samples examined using the reference Sysmex XN-9000. For each parameter, the HemoScreen minus the Sysmex XN-9000 test results are plotted against the mean of Sysmex XN-9000 and HemoScreen test results. The dotted lines indicate the 95% (1.96 SD) limits of agreement. The analytical bias is shown below each parameter. Thirty-one patients performed 161 home tests (2–11 tests per patient).

**Figure 3:**
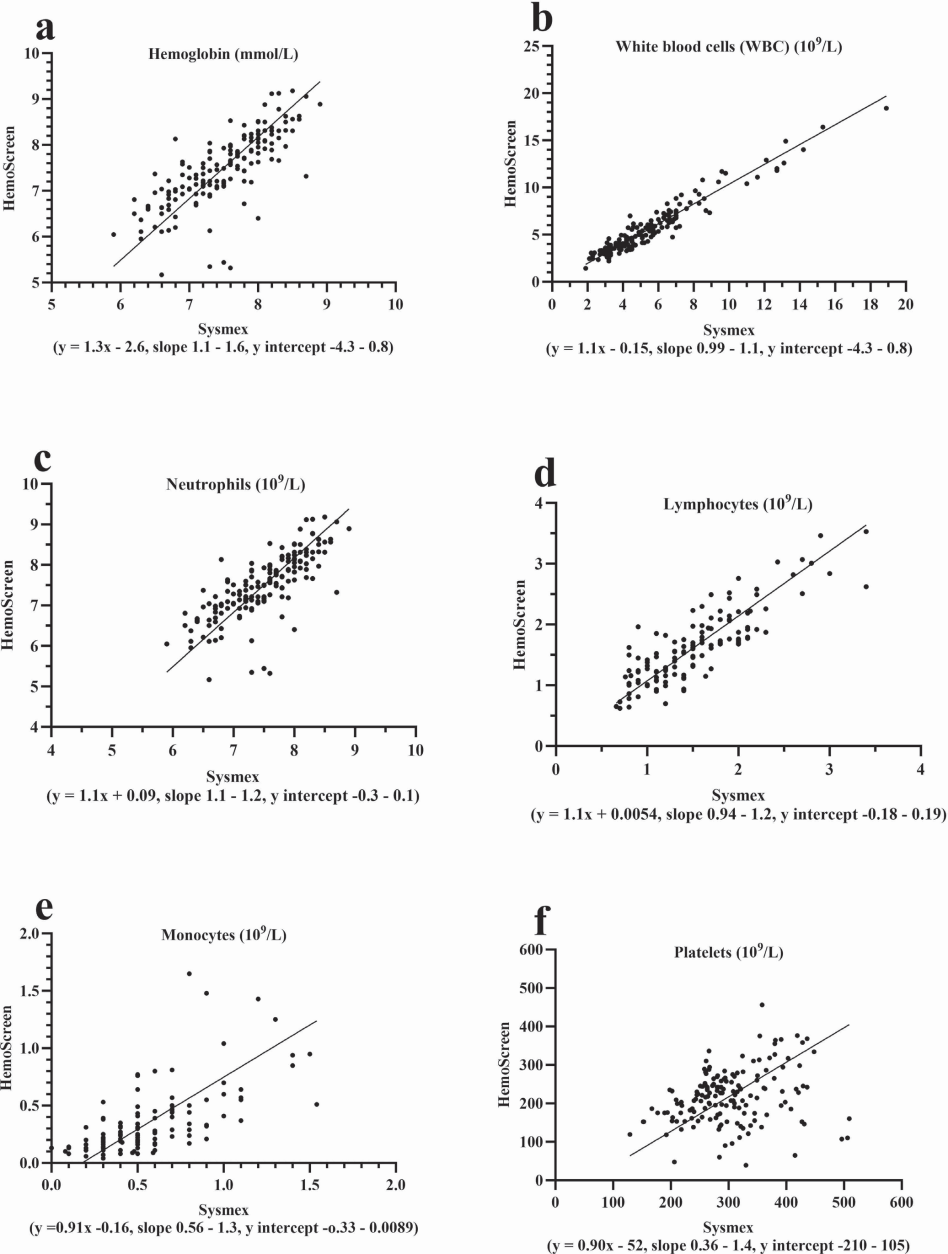
Deming regression analyses for patient collected capillary CBC analyzed using the HemoScreen compared with routine venous samples examined using the reference Sysmex XN-9000. Comparison of the patient’s capillary CBC self-testing analyzed by the HemoScreen with reference venous blood samples analyzed using the Sysmex XN-9000. Thirty-one patients performed 161 home tests (2–11 tests per patient).

All 39 patients learned to collect the capillary blood samples correctly (three had prior experiences in capillary blood collection) and to operate the HemoScreen instruments (Figure 1) without being assisted by a family member/co-habitant during self-testing. The median number of self-tests performed was 5 (range 2–11) and the median test error/failure rate was 0 (range 0–3) for the group of patients who completed the study. The correlation coefficient between the capillary HemoScreen CBC self-tests and the corresponding venous Sysmex reference CBC tests (Supplementary Table 2, Figure 2 and Figure 3) was a little lower than the instrument verification correlation between the HemoScreen venous and the Sysmex reference venous CBC tests (Supplementary Table 1). Despite this, the correlation, slope, intercept, and bias of the hemoglobin measurement and the leucocyte, neutrophil, lymphocyte, and monocyte counts were acceptable (supplementary information), and the capillary CBC test results were clinically usable and suitable for clinical decisions to be based on them. In contrast, the capillary platelet counts had a major bias of −52 10^9^/L, a slope of 0.90 and a correlation between capillary and venous CBCs of 0.26. Halfway through the study, the manufacturer PixCell upgraded the HemoScreen image recognition software; however, the impact on performance was limited, and the dataset was analyzed as one (Supplementary information Tables 2 and 3, Figures 1 and 2).

### Qualitative interviews

Data saturation was achieved when nine representative patients had completed the qualitative interviews, duration 55–95 min (Table 1). The number of self-tests conducted by each patient at the time of the interview ranged from three to 11 times depending on type of chemotherapy and progression of treatment (Table 1).

### Instrument usability

The participants that used the HemoScreen for self-testing found the instrument intuitive to operate and user-friendly. All the participants used the stepwise illustrative guide. None of the participants used the ‘how-to’ video, and only two of the nine interviewed used the phone hotline (one patient one time/the other twice) for solving technical problems. Overall, the patients found it meaningful to participate in the self-test study motivated by their belief that the interviews would contribute to breast cancer research and engage themselves more in their own treatment.

### Benefits of home-testing during treatment

All patients stressed the advantage of taking blood tests at home where treatment takes up most of their time and preventing unnecessary hospital visits would be valuable. The flexibility was particularly noted by women with children living at home. The patients emphasized the importance of the direct interaction with the physicians when interpreting the results and making decisions based on the home test. They underscored that the test results ‘cannot stand alone’.

### Becoming an active partner in the treatment

The qualitative findings found that self-testing at home could provide patients with an increased sense of autonomy, control, and ‘normality’. Many of the patients associated the frequent home tests, as a supplement to hospital treatment, with a feeling of security. Testing themselves and within minutes having the test results before their caregivers made the participants feel more involved and as a more ‘equal’ partner in the patient-caregiver relationship. Some also felt empowered and ‘in-control’ to prepare themselves for the outpatient visit (e.g. in the cases where treatment had to be postponed due to leuco-/neutropenia). Although none of the nine respondents had a healthcare background, they gained a good understanding of the CBC test, how to interpret the result, and expressed a desire to know more about how to interpret further the test results produced by HemoScreen testing to feel even more empowered. An important observation during the home interviews was that most patients placed the test device in their living room or other visible places, indicating their commitment and how important and a natural part of their everyday lives the device and self-testing had become for them.

## Discussion

The mixed methods approach in this proof-of-concept study allowed examining the quality of sampling and the factors influencing patient well-being when testing CBC at home. Knowledge of these factors is important for self-testing success since patient acceptance and involvement are pivotal [25]. The findings of the study provide a basis for the way forward for implementing self-monitoring of blood tests in oncology.

### Considerations regarding choice of WBC/CBC POCT instrument

For the study, we considered three POCT instruments: (1) HemoCue WBC (Radiometer, Copenhagen, Denmark) [17, 26], (2) the OLO CBC instrument (SightDx, Tel Aviv, Israel) [27] and the HemoScreen instrument (PixCell Medical, Yokne’am Illit, Israel). The HemoScreen was best suited and chosen for this study. The other instruments also fulfilled many of the technical and clinical needs regarding blood cell counting: All instruments used single analysis cartridges, were well suited for low volume intermittent use, could be operated by the patients themselves and could communicate with hospital IT systems. However, none of the instruments, including the HemoScreen are ideal for home monitoring; both the OLO CBC and the HemoScreen instruments are too big and heavy (5–10 kg) for being easily transportable and too expensive (>5.000 Euros). In contrast, the HemoCue WBC Diff instrument is well suited in size and in performance of the WBC counting, whereas it lacks the ability to measure hemoglobin and platelets. Cell phones are widely used and have cameras mounted that with an adapter supports the magnification needed for image recognition-based cell counting and hemoglobin measurement. Therefore, cell phones could be an attractive alternative to stand-alone CBC instruments. Furthermore, cell phone by nature also have built-in 4/5G connectivity that would support data transfer from the home to a hospital’s IT systems [28]. Such cell phone CBC count analysis-based solutions could in principle be similar to the Calprosmart system, which uses the cell phone’s camera for self-testing analyses and its connectivity for data transfer to the hospital [29].

### Analytical performance of the WBC/CBC POCT instruments

The analytical performance of the HemoScreen is close to that of standard hematology analyzers when testing venous blood samples [16, 30]. The observed slightly lower correlation between patients self-test and the reference sample presumably stems from a combination of slightly larger variation seen for capillary samples in general compared to that of venous samples even when collected by healthcare professionals [16, 20, 31] and that these samples furthermore were collected by the patients (this study and [17]). Proper sample collection is important for safe self-testing and venous samples undoubtedly produce the best test results; however, except for patients with permanent venous access, such samples are not easily obtainable. Furthermore, there are currently no reports on the safety of self-sampling from patients with a permanent central venous line. Therefore, self-testing will primarily have to rely on finger prick samples. The quality of POCT platelet count results has varied in other studies; some studies reported identical counts to venous samples, while others (including ours) reported lower values [21, 32]. The variation between platelet counts in venous and capillary blood samples is most likely caused by early activation of the hemostatic system [33]. There could also be a methodological issue as HemoScreen platelet counts have been reported to have negative bias (i.e. −20 to −10 10^9^/L even when testing venous samples) [16, 21]. Although patients whose platelet counts are underestimated, they are not facing any increased risk for bleeding. Underestimation can lead to an overestimation of the patients’ risk for severe bleedings. Even though this can result in unnecessary, costly, and time-consuming platelet transfusions, it will, however, not expose the patients to the dangers of major bleedings. Better platelet test results will depend on better sampling quality, which presumably will require better patient education and the development of improved capillary samplers that are easier for non-medical staff to operate.

### Need for seamless data transfer from the POCT instrument to the patient record

Even though all patients were able to transfer test results from their homes to the hospital records, the current method of taking pictures of the test results and transferring them was suboptimal. Self-testing and self-monitoring will become more attractive with wireless (e.g. 4/5G) transfer of the test results from instrument to the hospital patient record system. Hence, currently there is an unmet need to develop safe methods for automatically transferring data from the patients’ self-testing equipment to the hospital patient record systems.

### Patient empowerment through self-testing and the patient- healthcare staff interaction

The interviews demonstrated that the patients were motivated to self-test because of the convenience, ease of use and especially the feeling of empowerment, which mirrors findings in studies in other areas of medicine outside oncology (see supplementary information) [34]. The participants in the present study experienced flexibility in terms of treatment, active engagement, and a re-establishing of a sense of self and ‘normality’ gaining some kind of control of their own treatment similar to the previous finding [35].

Shared decision-making (e.g. ‘no decision about me without me’) has become the standard, including different degrees of patient and public involvement [36]. Until now, the healthcare professionals have performed the different blood tests and subsequently shared them with the patients before the shared decision making. Self-testing democratizes the production of laboratory test results and has the potential of facilitating a ‘care-transition’ that could bring the caregivers and the patients on a more ‘equal level’ [37, 38]. And since many healthcare systems are challenged by a staff shortage, there is an increasing demand for patients and their relatives to be involved in/take over responsibility for different aspects of the treatment [39]. Most of the interviewed patients in this study experienced a large degree of flexibility and autonomy being actively involved in their treatment. Other patients might have difficulties in identifying themselves in their new role as an ‘active’ patient (i.e. being involved in tests previously taken by health care professionals at the hospital) [40]. The patients and their relatives who do not have the necessary resources and qualifications needed for self-monitoring are especially at risk of finding the larger degree of patient responsibility disempowering [19]. Furthermore, introducing self-monitoring at home makes treatment more independent of time and space. While beneficial for some patients, the shift in the spatial dimensions of care has the risk that some patients no longer find their home as a ‘safe place’, free of sickness [41]. These factors make selection criteria and process of self-testing participants critical for good outcomes.

Finally, introducing self-monitoring in treatment also alters the interaction between healthcare staff and the patient in new ways through technology, and this will both affect and change the health care professionals´ needs, wishes, and concerns. It is therefore crucial to have an open dialogue about this and the reconfiguration of roles and responsibilities that increased patient involvement entails.

## Conclusion

We are in the early stages of self-monitoring/testing in oncology, with most studies focused on either the technical quality or the patient experience [16, 17, 42]. For self-testing to be successful, it is mandatory that the test results have the needed analytical quality, that the procedure is accepted by the patients and that they feel the setup is of value to them. The established setup fulfilled these criteria, and the combined qualitative and quantitative approach [43] gave a better understanding of the overall setup and its implications for the patients. In general, the patients quickly managed the self-testing and interpreting the results. When one of the participants was feeling ill, she, without the approval of the treating physician, self-tested outside the scheduled study tests, FN was quickly diagnosed and the patient was admitted in time to start antibiotic treatment. However, we also found that about one in four dropped out of the study for various reasons. It is therefore important that self-testing becomes a supplement and not a replacement of the current standard of care. The benefits and drawbacks of home monitoring in oncology should be balanced so that it does not lead to greater inequality in health.

## Supplementary Material

Home self-testing of complete blood counts in patients with breast cancer during chemotherapy: A proof-of-concept cohort study in e-oncology

## Data Availability

Restrictions apply to the availability of some or all data generated or analyzed during this study either because of the need to preserve patient confidentiality or because they were used under license. The corresponding author will, on request, detail the restrictions and any conditions under which access to some data may be provided.
