# Supplementary material for: Home self-testing of complete blood counts in patients with breast cancer during chemotherapy: A proof-of-concept cohort study in e-oncology
Source: Acta Oncol. 2024 Sep 18;63:41050. doi: 10.2340/1651-226X.2024.41050 (PMC11423696; doi:10.2340/1651-226X.2024.41050)
Supplement: Home self-testing of complete blood counts in patients with breast cancer during chemotherapy: A proof-of-concept cohort study in e-oncology [file AO-63-41050-s1.pdf]

Supplementary material has been published as submitted. It has not been copyedited, or typeset by Acta Oncologica

Supplementary Information

Power calculation

We assume the expected probability of success to po = 0.90. We decided that if the probability of success is p=0.70 (or less), then the HemoScreen may not be useful in a given context, i.e., with the given patient population included and the instruction provided.

The Null hypothesis of the study is Ho: p = po, and we want to test the alternative hypothesis Ha: p <po (one-tailed test) with a probability of 5% or less and a power of 80%, i.e. it is less than 20% probability that we overlook that only 70% or less can use the equipment correctly.

For calculation we use the program STATA version 15 power one-proportion 0.9 0.7, test (Wald) one-sided. The estimated necessary sample size is N = 33 patients.

Observation template: HemoScreen blood test, patients for certification at the outpatient clinic

Date:

Time (patient entering):

Patient SSN:

Time for self-test start:

1. In what order do the patient perform the elements of the test? Number the order of the single actions and how they are performed:
   ___________ Washes hands in warm water
   ___________ Press the Start-button
   ___________ Prepare the test materials
   ___________ Opens the package with sampler and cassette
   ___________ Desinfects finger, which finger?
   ___________ Massaging the desinfected finger
   ___________ Pricks the desinfected finger
   ___________ Discards the first drop of blood
   ___________ ”Milking” the finger in a downward position
   ___________ Collects the blood in the micro tube and turns it
   ___________ Pours the blood out on the paper
   ___________ Coolects the blood into the sampler
   ___________ Inserts the sampler into the test cassette
   ___________ Pushes ”Test” on the HemoScreen device

___________ Inserts the cassette into the HemoScreen device

Time stamp: _______________

___________ Takes two screenshot photos of the test results
___________ Oploads the photos to Min-SP
___________ Takes out the cassette and dispose it
___________ Pushes the button to turn out the device

Note the results of the self-test (Before the patient presses the botton)

WBC ________

RBC ________

HGB ________

PLT _________

NEU ________

LYM ________

MON ________

EOS _________

BAS _________

2. Could the patient perform the test without assistance? (Observers impression)
Yes /No / ____

3. Did the patient appear secure in performing the self-test? (Observers impression)
Yes / No / ____

4. Will the patient be able to perform a self-test at home? (Observers impression)
Yes / N / ____

Total score of the performance of self-test 1-10 (10 is the best possible performance), and other observations (optional)

Questionnaire for patients performing self-tests in the outpatient clinic

Introduction: This interview will last approx. 5 minutes. I would like to hear your immediate response to the self-test, which you have just performed.

1. How was your overall impression of the self-test?

   ____

2. Was anything particularly challenging? If yes, why?

   ____

3. How was your overall impression of the test instructions?

   ____

4. Did you feel prepared to perform the self-test after the instructions?

   ____

5. After the instructions, do you feel prepared to perform the self-test in your own home? If No, why not?

   ____

6. Could you imagine doing a self-test in your own home, if it could save you from unnecessary trips to the clinic? If No, why not?

   ____

7. Do you already have earlier experiences with performing blood self-tests?

   ____

Mange tak for din deltagelse (many thanks for your participation)

Time stamp (patient leaves the room) ___________

Observation template: HemoScreen blood test, patients

Interview guide

Thank you for participating in this interview. There are no right or wrong answers, but your experiences and opinions are important to understand better, how we can best help other patients in their everyday life. I come from a different unit at the hospital and have

no personal interest in HemoScreen, I am only interested in your experiences. This interview is expected to last approximately one hour. The interview is recorded digitally and is subsequently printed out on paper. This material is anonymized, so you are completely anonymous. Your statements will in no way influence your course of treatment.

Timeline

Please place the following events on the timeline:

- When were you diagnosed with cancer? (Possibly also if there have been several courses)
- When did your course of treatment begin? What types of treatment courses?
- When were you introduced to the HemoScreen at the hospital?
- When did you do your first self-test at home? (second, third etc...)
- How many times have you used the HemoScreen device?
- When did you receive your first treatment at the hospital after self-testing?
- At what point of the treatment process are you now?

The patient's use of the HemoScreen device

- What motivated you to join this research project?
- How has your experience of taking blood tests yourself at home been?
- Do you have previous experience with needles and blood tests or the like?
- Did you experience any challenges with it? If so, which ones?

The patient's use of Epic app "MinSP"

- How did taking pictures and sending via the MinSP app work?
- Did you experience any challenges with the app?
- How would you feel if the HemoScreen device itself sent your data to the hospital?
- Or if you could send them to the hospital directly via the device?

The patient's use of aid

- How did you find the nurse's instructions for the device?
    - Did you still remember the nurse's introduction when you first had to self-test at home?
    - How did you feel at that moment?
- Did you need help doing the blood tests at home? If so, for what?
- Did you use the quick guide when doing self-tests? (Find the guide and look at it together)
    - Which parts have you used, and which have you not?
    - Have you changed the order?
    - Did you miss anything in the guide?
    - Anything that could be done differently?
    - Have you used the guide in other situations? (E.g. showed it to someone)
- Have you used the video instructions? (Watch the video together)
    - Where have you seen it?
    - How did you use it?
- Did you use the Hotline for help?

    - What did you use it for and how was it?
- Have you received any help from your relatives?
    - If so, who and for what?

Feeling safe

- How do you feel about reading the test results on the screen? Are you looking at them yourself?
    - Do you know the meaning of the results?
    - Is it different to get the results this way?
    - Did you keep track of your blood measurements before you got a HemoScreen device at home?
    - Have you tried to get a result of a blood test which meant that you could not receive your planned chemotherapy afterwards? What was it like?
- Do you feel safe using the device yourself at home?
- Were there times when you didn't feel comfortable about it?

Pros and Cons of blood self-tests at home (implementation)

- What would it mean to you not to have to drive to the hospital every time for blood tests?
    - What difference would it make in your everyday life?
    - What difference would it make in your course of treatment?
- Do you think it would be an advantage for other patients to be able to take blood tests themselves at home?
- What kind of challenges do you think other patients could have with it?

Co-creation element

- To make self-testing suited for you in the best possible way, what would you need?
- How could the device be improved?
- How could the app be improved?
- How could the process be improved?

Side effect scheme

- Did you fill out the side effect scheme?
- How was your experience with the scheme?
- Did it cover all your side effects?
- If you have had side effects of your cancer treatment, have they influenced your use of the device?

For possible relatives:

- How is your experience with blood tests at home? Are you comfortable with it?
- Did it affect your everyday life? How?
- What difference would it make to you, if she (the patient) did not have to drive to the hospital for blood tests?

Final remarks

Is there anything I' have forgotten or anything you would like to add?

Performance Criteria

Venous HemoScreen vs venous Sysmex CBC method comparison performance criteria

The performance of the HemoScreen when analyzing venous CBCs has previously been examined [1-7]. Based on the these results the follow criteria were set for CBC not including eosinophil and basophil granulocytes: A correlation >0.95, a Deming regression slope between 0.95 – 1.05, an intercept -0.3 – 0.3 mmol/L for hemoglobin, -0.3 – 0.3 10^9/L for WBC and -10 – 10 10^9/L for platelets. The bias should < 5% for hemoglobin, <10% for WBCs > 1 10^9/L and for WBC < 1.0 10^9/L an absolute bias <0.2 10^9/L. For platelets the criteria were 5% for platelets >50*10^9/L and an absolute limit = 5*10^9/L for platelets ≤50*10^9/L. The instruments passed met criteria (supplementary Table 1)

Supplementary Table 1. Technical verification of the two HemoScreen instruments

The performance of the HemoScreen instrument was verified by parallel analysis of 30-35 venous blood samples analyzed using a Sysmex XN-9000 reference instrument. To verify the performance of the HemoScreen the results from an earlier verification study are included for reference. [7]

**Parameter**	**N**	**Correlation coeff**	**Slope**	**Intercepts**
**Hgb (mmol/L)**	**34**	**0.95 (0.90 - 0.97)**	**1.0 (0.89 – 1.2)**	**-0.29 (-1.3 – 0.69)**
Ref [7] (mmol/L)	30	0.980 (0.96 - 0.99)	0.98 (0.9, 1.06)	0.076 (−0.56 – 0.66)
**WBC (10^9/L)**	**32**	**0.99 (0.97 – 0.99)**	**1.0 (0.88 – 1.1)**	**-0.19 (-0.84 – 0.46)**
Ref [7] (10^9/L)	30	0.97 (0.94 - 0.99)	0.81 (0.74 - 0.89)	0.65 (−0.26, 1.6)
**NEU (10^9/L)**	**29**	**0.99 (0.99 – 1.0)**	**0.98 (0.94 - 1.0)**	**0.26 (0.01 – 0.51)**
Ref [7] (10^9/L)	30	0.97 (0.94 - 0.99)	0.82 (0.74 - 0.90)	0.31 (−0.47 - 1.1)
**LYMP (10^9/L)**	**29**	**0.97 (0.94-0.99)**	**0.83 (0.72 – 0.95)**	**027 (0.088 - 0.46)**
Ref [7] (×10^9/L)	30	0.98 (0.95 - 0.99)	0.94 (0.86 - 1.0)	0.021 (−0.20 - 0.16)
**MONO (10^9/L)**	**128**	**0.64 (0.53 – 0.74)**	**0.91 (0.56 – 1.3)**	**-0.16 (-033 – 0.0089)**
Ref [7] (×10^9/L)	30	0.82 (0.65 - 0.91)	0.87 (0.63 - 1.1)	0.031 (−0.21 - 0.15)
**PLT (10^9/L)**	**35**	**0.99 (0.97 – 0.99)**	**1.0 (0.88 – 1.1)**	**10 (-9 – 30)**
Ref [7] (×10^9/L)	30	0.99 (0.97 - 0.99)	1.09 (1.0 - 1.2)	10 (−14 - 34)

Software upgrade: Before (old software) and after (new software).

The impact of software upgrade on the actual measured values was without clinical importance and the CBC data and presented in the paper and one set of data. However, because the data were collected in real time, the upgrade in the image recognition software divided the dataset; The before the upgrade/the "old" software and after the upgrade/the "new" software. To examine the effects of the software, upgrade the dataset was there divided in two and reanalyzed. The software upgrade overall improved performance of the instrument (Suppl. Figure 1). There were no hemoglobin values below 5 mmol/L. Analysis of hemoglobin 5-7 mmol/L showed better performance (slope 1.5/intercept -3.7 before vs 1.2/-1.3) after the software upgrade (Suppl. Table 2 and Suppl Figure 1). The number of observations after the upgrade was too small to draw firm conclusions regarding the detection of neutropenia (Suppl. Table 3). The capillary HemoScreen test underestimated the monocyte counts and this was unaffected by the software upgrade (Suppl. Table 2 and Suppl. Figure 1). However, the capillary platelet counts analyzed with the old software performed poorly with a slope of 0.48, an intercept of 55 10^9/L and a correlation of 0.13. The software upgrade improved the performance (slope 0.99, though the intercept was -51 10^9/L and the correlation 0.57). This bias could be suggestive of loss of platelets during collection and count process.

In summary, the capillary platelet counts still underestimated the true counts. However, since none of the patients had platelet counts <50 10^9/L, the performance of the capillary platelet count for the thrombocytopenic (<50 10^9/L) patients, who are at high risk for bleeding, could not be assessed. Lastly, the software upgrade did not change the white blood cell count (WBC) and lymphocyte count with range tested in this study.

Overall, the software upgrade had little impact on the actual measured values partly for several reasons. First the WBC, NEU, LYMF and EOS counts well with the old software and hence the improvement was minor. For the Hemoglobin, most of the test results were within the refence interval and in this range the impact was small. However, it seems as though this there might be improved quality when testing anemia, but there few data to examine this properly. The same seems to be case for platelets but again there were too few measurements from patients with severe thrombocytopenia to allow for an evaluation of clinical impact on the clinical impact of the upgrades effect on the platelet counts.

Capillary HemoScreen vs venous Sysmex CBC method comparison performance criteria

The performance of the HemoScreen when analyzing venous CBCs has previously been examined [1,2,5,8]. Based on the these results the follow criteria were set for CBC not including eosinophil and basophil granulocytes: A correlation >0.90, a Deming regression slope between 0.90 – 1.1, an intercept -0.5 – 0.3 mmol/L for hemoglobin, -0.3 – 0.3 10^9/L for WBC and -10 – 10 10^9/L for platelets. The bias should < 5% for hemoglobin, <10% for WBCs > 1 10^9/L and for WBC < 1.0 10^9/L an absolute bias <0.2 10^9/L. For platelets the criteria were 10% for platelets >50*10^9/L and an absolute limit = 10*10^9/L for platelets ≤50*10^9/L. The instruments passed met criteria (supplementary Table 1) except platelets as discussed in the paper.

Supplementary. Table 2. Performance of capillary CBC testing performed by patients.

Comparison of the patient's capillary CBC self-testing analyzed by the HemoScreen with reference venous blood samples analyzed using the Sysmex XN-9000. The HemoScreen cell analysis software was updated halfway through the study. For each parameter three set of analyses were done: 1) "Bold" = All data irrespective whether it was generated before or after the software update, 2) Old = data generated before the update and 3) New = data generated after the update.

Parameter	N	Correlation coeff	Slope	Intercepts
**Hgb (mmol/L)**	**161**	**0.76 (0.69 - 0.82)**	**1.3 (1.1 - 1.6)**	**-2.6 (-4.3 - -0.84)**
Old (mmol/L)	99	0.72 (0.61 - 0.80)	1.5 (1.2 - 1.8)	-3.7 (-6.3 - -1.1)
New (mmol/L)	62	0.81 (0.70 - 0.88)	1.2 (0.90 - 1.5)	-1.3 (-3.5 - 0.87)
**WBC** (10^9/L)	**161**	**0.96 (0.95 – 0.97)**	**1.1 (0.99 – 1.1)**	**-0.15 (-0.47 – 0.16)**
Old (10^9/L)	98	0.97 (0.95 – 0.98)	1.0 (0.96 – 1.1)	-0.14 (-0.48 – 0.20)
New(10^9/L)	63	0.94 (0.91 – 0.96)	1.1 (0.89 – 1.2)	-0.14 (-1.1- 0.86)
**NEU** (10^9/L)	**157**	**0.94 (0.92 – 0.96)**	**1.1 (1.1 – 1.2)**	**-0.092 (-0.27 – 0.087)**
Old (10^9/L)	97	0.97 (0.96 – 0.98)	1.1 (1.0 – 1.2)	-0.038 (-0.22 – 0.15)
New (10^9/L)	67	0.87 (0.79 – 0.92)	1.2 (1.0 – 1.4)	-0.28 (-0.89 – 0.34)
**LYMP** (10^9/L)	**131**	**0.86 (0.80-0.90)**	**1.1 (0.94 – 1.2)**	**0.01 (-0.18 - 0.19)**
Old (10^9/L)	77	0.86 (0.79 – 0.91)	1.1 (0.98 – 1.2)	-0.07 (-0.25 - 0.12)
New (10^9/L)	54	0.80 (0.68 – 0.88)	0.96 (0.70 – 1.2)	0.20 (-0.22 - 0.62)
**MONO** (10^9/L)	**128**	**0.64 (0.53 – 0.74)**	**0.91 (0.56 – 1.3)**	**-0.16 (-033 – 0.0089)**
Old (10^9/L)	68	0.63 (0.46 – 0.75)	0.91 (0.42 – 1.4)	-0.13 (-0.36 – 0.10)
New (10^9/L)	60	0.69 (0.52 – 0.80)	0.88 (0.38 – 1.4)	-0.18 (-0.46 – 0.065)
**PLT** (10^9/L)	**160**	**0.26 (0.11 – 040)**	**0.90 (0.36 – 1.4)**	**-52 (-210 – 105)**
Old (10^9/L)	98	0.13 (-0.065 – 0.32)	0.48 (-0.40 – 1.4)	55 (-204 – 314)
New (10^9/L)	62	0.57 (0.37 – 0.71)	0.99 (0.50 – 1.5)	-51 (-199 – 96)

Supplementary Figure 1. Deming regression analyses for patient collected capillary CBC analyzed using the HemoScreen compared with routine venous samples examined using the reference Sysmex XN-9000.

Comparison of the patients capillary CBC self-testing analyzed by the HemoScreen with reference venous blood samples analyzed using the Sysmex XN-9000. The HemoScreen cell analysis software was updated halfway through the study. For each parameter three set of analyses were done: 1) HS total = All data irrespective whether it was generated before or after the software update, 2) Old = data generated before the update and 3) New = data generated after the update.

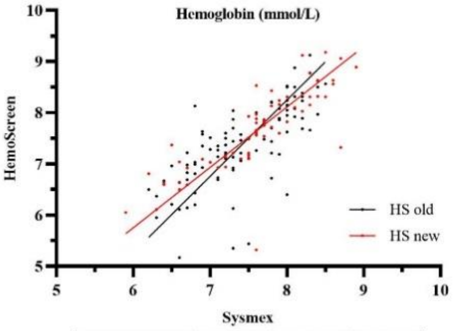

	HS total	HS new	HS old
Slope	1.3	1.2	1.5
Y-intercept	-2.6	-1.3	-3.7

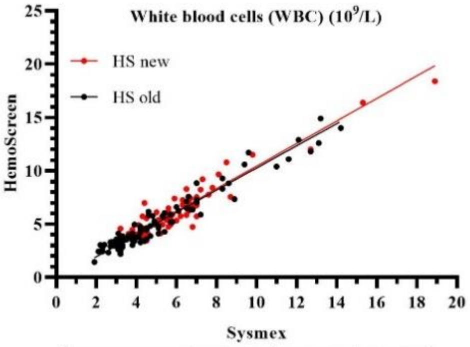

	HS total	HS new	HS old
Slope	1.1	1.1	1.0
Y-intercept	-0.15	-0.14	-0.14

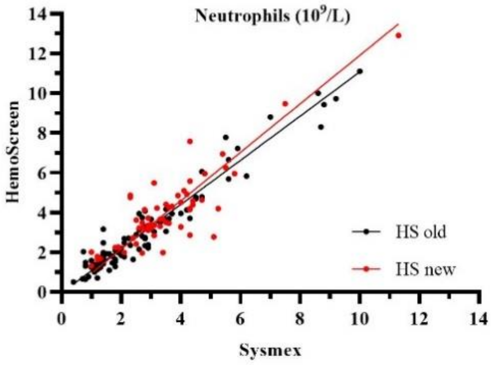

	HS total	HS new	HS old
Slope	1.1	1.2	1.1
Y-intercept	-0.092	-0.28	-0.038

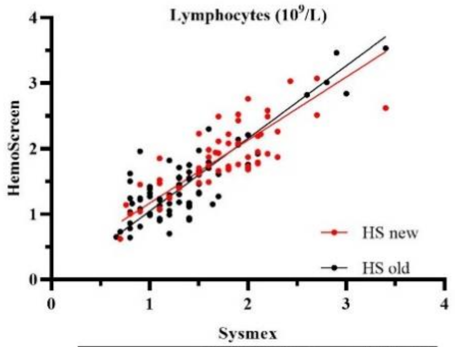

	HS total	HS new	HS old
Slope	1.1	0.96	1.1
Y-intercept	0.0054	0.20	-0.065

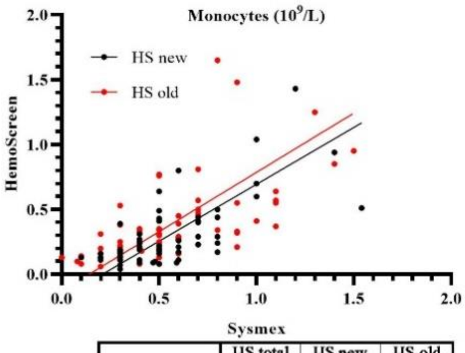

	HS total	HS new	HS old
Slope	0.91	0.88	0.91
Y-intercept	-0.16	-0.18	-0.13

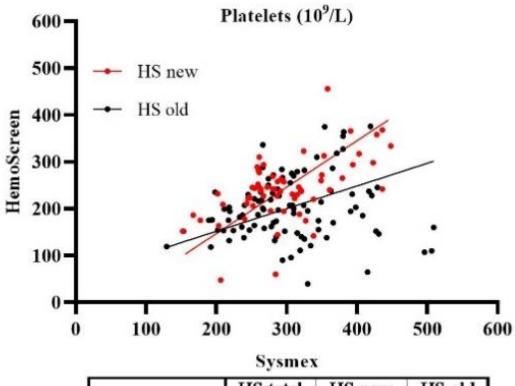

	HS total	HS new	HS old
Slope	0.90	0.99	0.48
Y-intercept	-52	-51	55

Supplementary Figure 2 Deming regression and Bland-Altman analyses of hemoglobin < 7 mmol/L and neutrophil counts < 3 10^9/L.

Bland-Altman: For each parameter, the Sysmex XN-9000 minus the HemoScreen test result is plotted against the mean of Sysmex XN-9000 and HemoScreen test results. The dotted lines indicate the 95% (1.96 SD) limits of agreement. The analytical bias is shown below each parameter.

Deming regression analyses of the capillary CBC self-testing analyzed by the HemoScreen with reference venous blood samples analyzed using the Sysmex XN-9000 for hemoglobin < 7 mmol/L (Panel C) and neutrophil counts < 3 10^9/L.

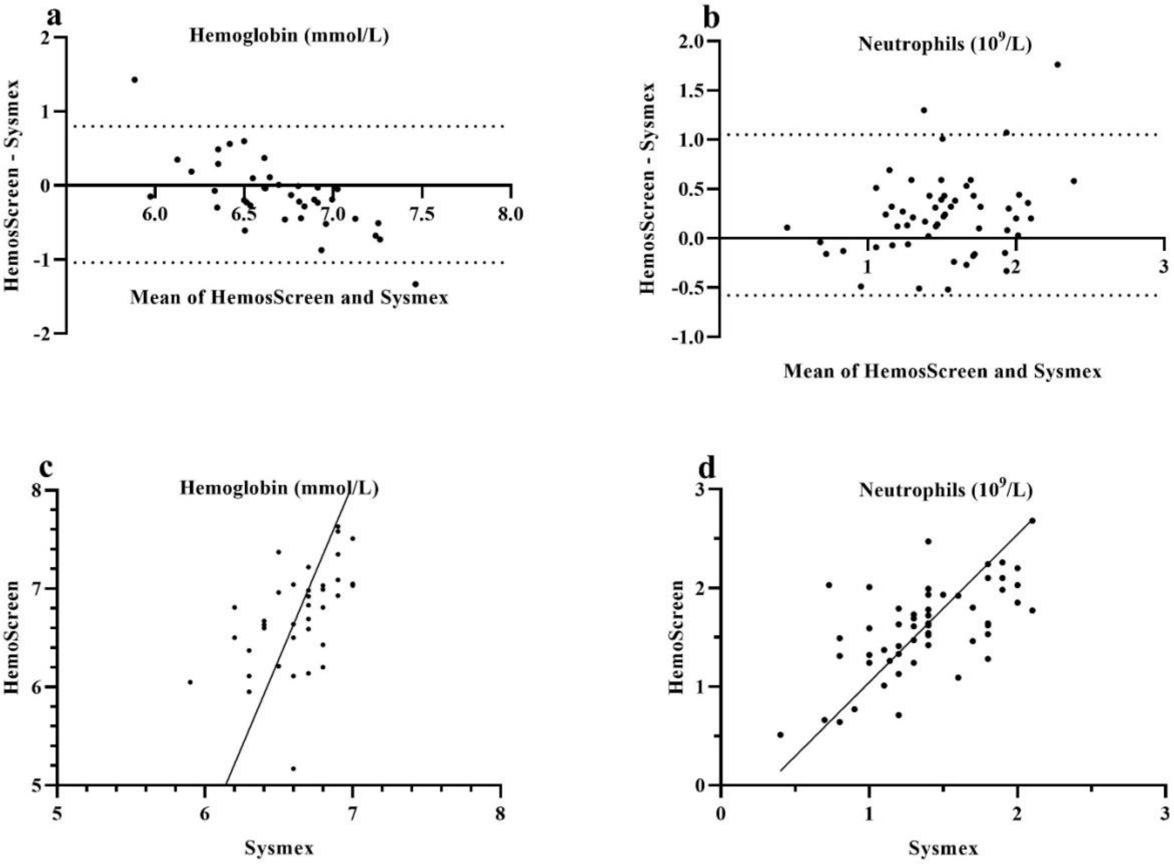

Supplementary Table 3 Low hemoglobin (<7 mmol/L) and low neutrophil counts (<2 10^9/L)

Comparison of patient's low capillary hemoglobin levels (< 7mmol/L) and low neutrophil counts (<2 10^9/L) self-testing analyzed by the HemoScreen with reference venous blood samples analyzed using the Sysmex XN-9000. : 1) "Bold" = All data irrespective whether it was generated before or after the software update, 2) Old = data generated before the update and 3) New = data generated after the update.

Parameter	N	Correlation coeff	Slope	Intercepts
**Hgb (mmol/L)**	**39**	**0.52 (0.25 - 0.72)**	**3.6 (0.94 – 6.2)**	**-17 (-34 - 0.65)**
Old (mmol/L)	25	0.55 (0.20 - 0.78)	5.1 (1.4 - 8.7)	-27 (-52 - -2.3)
New (mmol/L)	14	0.65 (0.19 - 0.88)	1.3 (0.23 - 2.5)	-1.3 (-9.4 – 5.2)
**NEU** (10^9/L)	**57**	**0.58 (0.38 – 0.73)**	**1.5 (0.86 – 2.1)**	**-0.46 (-1.3 – 0.39)**
Old (10^9/L)	50	0.57 (0.37 – 0.74)	1.5 (0.79 – 2.2)	-0.52 (-1.5 – 0.44)
New (10^9/L)	7	0.77 (0.05 – 0.96)	0.92 (0.15 – 1.7)	0.61 (-0.54 – 1.8)

Supplementary Table 4 Clinical decision-making Intervals for Hemoglobin, neutrophils and platelets.

The clinical concordance of the test results was examined by dividing the hemoglobin, absolute neutrophil counts and platelet counts into five clinical groups using the limits defined below.

Parameter	Severely low	Moderately low	Mildly low	Reference interval	High
Hemoglobin (mmol/L)	< 4.7	4.7 – 6.0	6.0 - 7.3	7.3 - 9.5	> 9.5
Neutrophils (10^9/L)	< 0.5	0.5 – 0.9	1.0 – 1.5	1.6 - 5.9	> 5.9
Platelets (10^9/L)	<20	20-50	50 – 145	145 - 390	> 390

The intervals were for hemoglobin were based on the Danish transfusion guideline and the Danish reference interval for hemoglobin [9]. The intervals for neutrophil risk classification were based on [1,10] and the Danish reference intervals for neutrophils for adults [9]. The levels for the platelet count based risk stratification were based guidelines for platelet transfusions [11] and on the Danish reference intervals for platelets for adults.

Supplementary Figure 3

The results were defined as concordant if the test results (low (severe, moderate, severe), within the reference interval or above the reference interval) was classified the same way by both the HemoScreen POCT method and Sysmex reference method, while a discrepancy in classification was considered a discordant result. Mildly discordant if the results only differed one class (Supplementary Table 4) indicated by light gray shading, panel A, C and E and severely discordant if the test results differed two

classes or more indicated by dark gray shading, panel A, C and E. The values measured for hemoglobin, neutrophil count or platelet count by the reference method (Sysmex) and the POCT (HemoScreen) are plotted in the corresponding panels (B; D and F). The clinical decision limits are also plotted (the dotted lines) in the panels (B, D, and F). The number concordant results reported by HemoScreen POCT instrument compared to Sysmex reference samples test results were calculated, shown by the numbers in each square panel A, C and E.

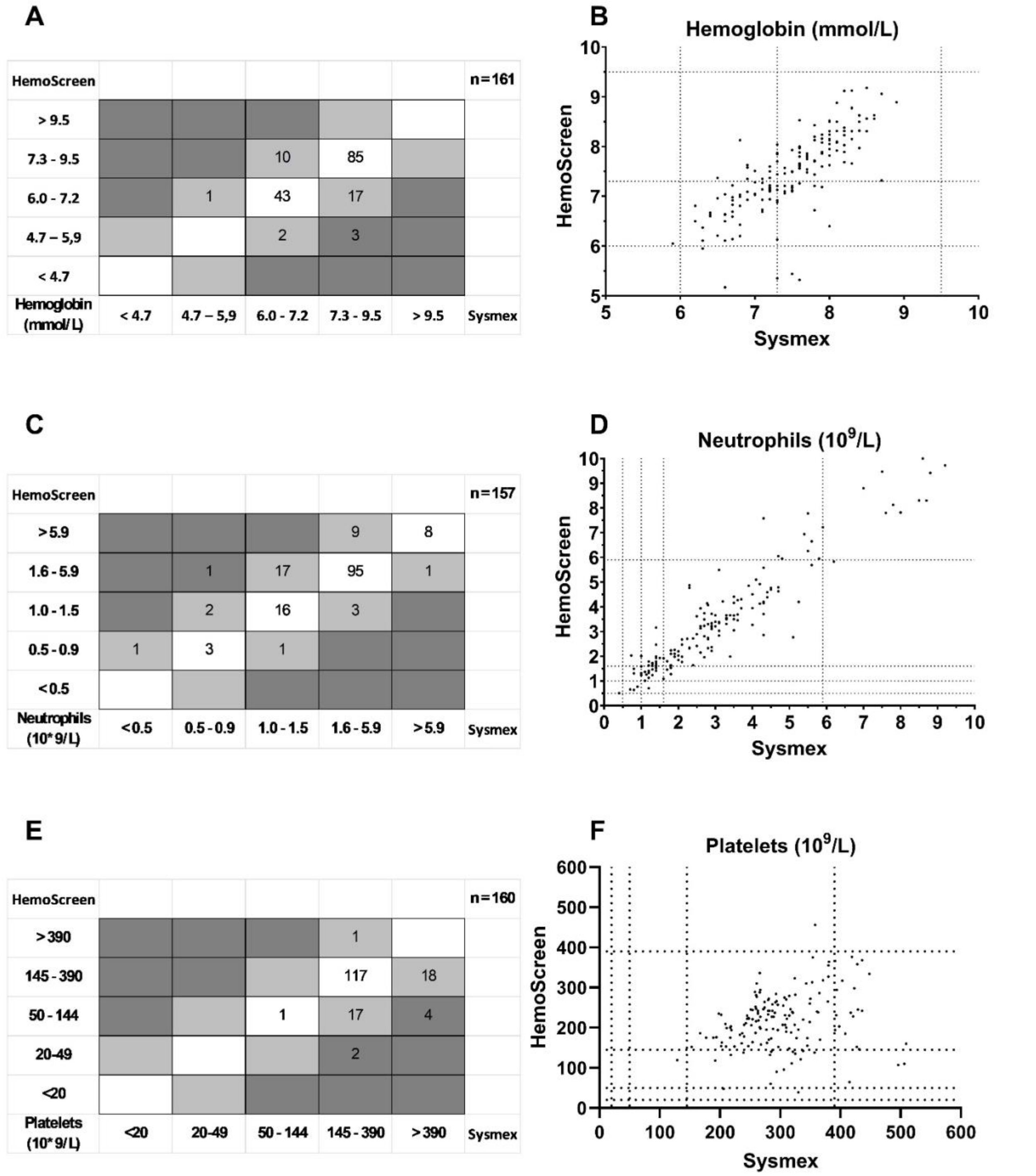

Supplementary material has been published as submitted. It has not been copyedited, or typeset by Acta Oncologica

Supplementary Table 5

The overall concordance and discordance were calculated by adding the number of all the concordant, the number mild discordant and the number of severe discordant number of samples for each parameter and calculating the % for each parameter out of the total.

	Concordant	Mild discordance	Severe discordance
**Hemoglobin**	**79%**	**19%**	**2%**
**Neutrophils**	**77%**	**22%**	**1%**
**Platelets**	**74%**	**22%**	**4%**

This showed that between 74-79% of results were concordant, 19-22% were mild discordant and 1-4% were severely discordant. The clinical bias also differed between the different parameters. Eighty-six % of the 23% discordant neutrophil count overestimated the neutrophil counts. In contrast two-thirds of the 21% discordant hemoglobin test underestimated the hemoglobin level and most significantly ninety-eight % of the discordant platelet test underestimated the platelet count. The positive bias regarding the neutrophil counts and the negative bias for the hemoglobin and platelet counts parallels earlier observations [1,2,5]. The bias was more pronounced when comparing capillary POCT samples with venous reference samples than when comparing venous POCT sample results with matching venous reference samples. The poorer performance of the capillary samples is indicative of challenges with the self-collected capillary samples. The main deviations observed are the overestimation of the neutrophil counts which was also found by Atkins et al. [8] but not by Maynard et al. [1]. Overestimating the neutrophil count could lead to an underestimation of their risk for infections and their bone marrow's ability to handle high -dose chemotherapy. In contrast the underestimation of hemoglobin and platelet counts could lead to unnecessary transfusions. Though this can burden both the patients and the healthcare system the risk for patients is very low. However, under all circumstances our findings points to the need for more focus on teaching and practicing proper capillary sample collection before home self-testing can be introduced on a broader scale.

References

1. Maynard RD, Funk T, Harrill W, Jin J, Smith D, Smith G, et al. Analytical Performance Evaluation of Three Point-of-Care CBC Analyzers for Management of Clozapine Therapy in Ambulatory Psychiatry Clinics. J Appl Lab Med 2023; 8: 1028-1041. https://doi.org/10.1093/jalm/jfad050.

2. Atkins M, McGuire P, Balgobin B, Desouza N, Taylor D. Haematological point of care testing for clozapine monitoring. J Psychiatr Res 2023; 157: 66-71. https://doi.org/10.1016/j.jpsychires.2022.11.027.

3. Linko-Parvinen AM, Keranen K, Kurvinen K, Tienhaara A. HemoScreen hematology analyzer compared to Sysmex XN for complete blood count, white blood cell differential, and detection of leukocyte abnormalities. EJHaem 2022; 3: 1126-1134. https://doi.org/10.1002/jha2.566.

4. Dickerson WM, Yu R, Westergren HU, Paraskos J, Schatz P, Tigerstrom A, et al. Point-of-care microvolume cytometer measures platelet counts with high accuracy from capillary blood. PLoS One 2021; 16: e0256423. https://doi.org/10.1371/journal.pone.0256423.

5. Kristian Kur D, Thogersen D, Kjeldsen L, Friis-Hansen L. The HemoScreen hematology point-of-care device is suitable for rapid evaluation of acute leukemia patients. Int J Lab Hematol 2021; 43: 52-60. https://doi.org/10.1111/ijlh.13330.

6. Larsson A, Smekal D, Lipcsey M. Rapid testing of red blood cells, white blood cells and platelets in intensive care patients using the HemoScreen point-of-care analyzer. Platelets 2019; 30: 1013-1016. https://doi.org/10.1080/09537104.2018.1557619.

7. Ben-Yosef Y, Marom B, Hirshberg G, D'Souza C, Larsson A, Bransky A. The HemoScreen, a novel haematology analyser for the point of care. J Clin Pathol 2016; 69: 720-725. https://doi.org/10.1136/jclinpath-2015-203484.

8. Atkins M, McGuire P, Balgobin B, Patel P, Taylor D. Using a fingerstick test for haematological monitoring in patients treated with clozapine. Ther Adv Psychopharmacol 2021; 11: 20451253211000865. https://doi.org/10.1177/20451253211000865.

9. Nordin G, Martensson A, Swolin B, Sandberg S, Christensen NJ, Thorsteinsson V, et al. A multicentre study of reference intervals for haemoglobin, basic blood cell counts and erythrocyte indices in the adult population of the Nordic countries. Scand J Clin Lab Invest 2004; 64: 385-398. https://doi.org/10.1080/00365510410002797.

10. Connelly JA, Walkovich K. Diagnosis and therapeutic decision-making for the neutropenic patient. Hematology Am Soc Hematol Educ Program 2021; 2021: 492-503. https://doi.org/10.1182/hematology.2021000284.

11. Neunert C, Terrell DR, Arnold DM, Buchanan G, Cines DB, Cooper N, et al. American Society of Hematology 2019 guidelines for immune thrombocytopenia. Blood Adv 2019; 3: 3829-3866. https://doi.org/10.1182/bloodadvances.2019000966.
